# The Russia–Ukraine war disproportionately threatens the nutrition security of developing countries

**DOI:** 10.1007/s43621-022-00112-8

**Published:** 2022-11-17

**Authors:** Zhongci Deng, Cai Li, Zhen Wang, Ping Kang, Yuanchao Hu, Haozhi Pan, Gang Liu

**Affiliations:** 1 College of Resources and Environment, Huazhong Agricultural University, Wuhan, 4300770 China; 2 Interdisciplinary Research Center for Territorial Spatial Governance and Green Development, Huazhong Agricultural University, Wuhan, 430070 China; 3 Plateau Atmosphere and Environment Key Laboratory of Sichuan Province, School of Atmospheric Sciences, Chengdu University of Information Technology, Chengdu, 610225 China; 4 School of Resources and Environmental Sciences, Wuhan University, Wuhan, 430079 China; 5 School of International and Public Affairs, China Institute for Urban Governance, Shanghai Jiao Tong University, 800 Dongchuan Rd., Shanghai, 200240 China; 6 Department of Green Technology, SDU Life Cycle Engineering, University of Southern Denmark, 5230 Odense, Denmark

**Keywords:** Russia–Ukraine war, Global nutrition security, Undernourishment

## Abstract

While the ongoing Russia–Ukraine war threatens global nutrition security, the magnitude and extent of its impact remain underexamined. Here we show that, with the lowest level of war duration, severity, sanction, and countries involved, the direct and indirect impacts of the war and sanctions could newly place 67.3 million people (roughly equals the total population of France) in undernourishment and 316.7 million people (roughly equals the total population of Bangladesh and Russia) suffering from extreme national food insecurity. Approximately 95% of the affected population are from developing countries, highlighting the vulnerability of food supply in these countries. Both the undernourished population and its inequality across countries will substantially grow, if war duration and severity increase. If the war is prolonged to early 2024, future agricultural growth cannot fully offset the negative impacts, and global hunger will still very likely exacerbate. We conclude that targeted measures should be placed in developing countries and their vulnerable populations to reconstruct a just, healthy, and environmentally sustainable food system.

**Supplementary Information:**

The online version contains supplementary material available at 10.1007/s43621-022-00112-8.

## Introduction

International and regional trade benefit food access by affecting prices, food availability, and agricultural labour [1, 2]. The liberalization of food trade in the context of globalization is considered to be an effective way to improve the competitiveness and productivity of the country's agricultural sector and to improve the country's food security [3, 4]. Meanwhile, some studies also point out that regional food trade can not only alleviate food shortages, but also help mitigate climate change [1]. The global market is an important source of food supply, especially for countries where domestic food production is limited by factors such as production technology [5] or agro-climatic [3, 6]. Currently, most Asian and African countries are still not self-sufficient, and the impediment of global and regional food trade will undoubtedly have an severe impact on food security in these countries [7]. However, the Russia–Ukraine war and associated sanctions have severely disrupted food production and trade [8]. As the war progresses, labour shortages and the coupled transportation restrictions and lower productivity in Ukraine and Russia have reduced agricultural production and limited the trade capacity of both countries [9]. Ukraine and Russia are the world’s leading food exporters; at least 26 countries, including Somalia, Senegal, and Egypt, rely on imports from one or both of these countries for 50–100% of their wheat consumption [10], which may already be suffering from severe food insecurity [11]. In addition, the war and consequent economic collapse also restricted access to agricultural inputs, greatly reducing agricultural productivity in warring countries (The civil war in Syria has reduced domestic food production by about 30%) [12]. Moreover, the supply chain disruption has caused a spike in food prices, thus prompting fear of social unrest across the globe. The negative effects of the war between Russia and Ukraine, and sanctions against them are expected to spill over to other countries, making the war another global nutrition-security-threatening event following COVID-19 [13, 14]. A few studies have described the food security impacted by the war by affecting the price and social stability, and analyzed the possible measures counteracting the threats. Although the nutritional threat posed by the war is recognized globally, a robust and quantitative assessment of the magnitude and heterogeneity of its impact on other countries is still lacking [15–17]. The war will impact the prevalence of undernourishment (PoU) and food supply directly through the reduction of food exports from Russia and Ukraine, and indirectly through reduced agricultural production in many global regions due to international sanctions, and labour and material shortages. Here, we built an extended adaptive multi-regional input–output (E-AMRIO) model [18] to assess both the direct and indirect impacts of the Russia–Ukraine war on the global prevalence of undernourishment (PoU, the proportion of undernourished people, the main indicator used by FAO to assess global hunger).

This model incorporates the latest available global supply chain data (GTAP10) [19], and accounts for interactions between supply and demand at the sectoral level (e.g. flour production requires wheat, which is also affected by flour demand). Compared to models with annual time steps (such as computable general equilibrium models), this E-AMRIO model has the advantage of a weekly time step, which is particularly useful for evaluating short-term contingencies [19–21]. We aim to reveal the impact of different war trajectories on the nutritional security of different countries by using granular scenario designs. The results will help to identify the potential food insecure population across the globe and support tailored decision-making to alleviate additional hunger caused by the war.

## Methods

### Impact assessment based on the E-AMRIO model

**Fig. 1:**
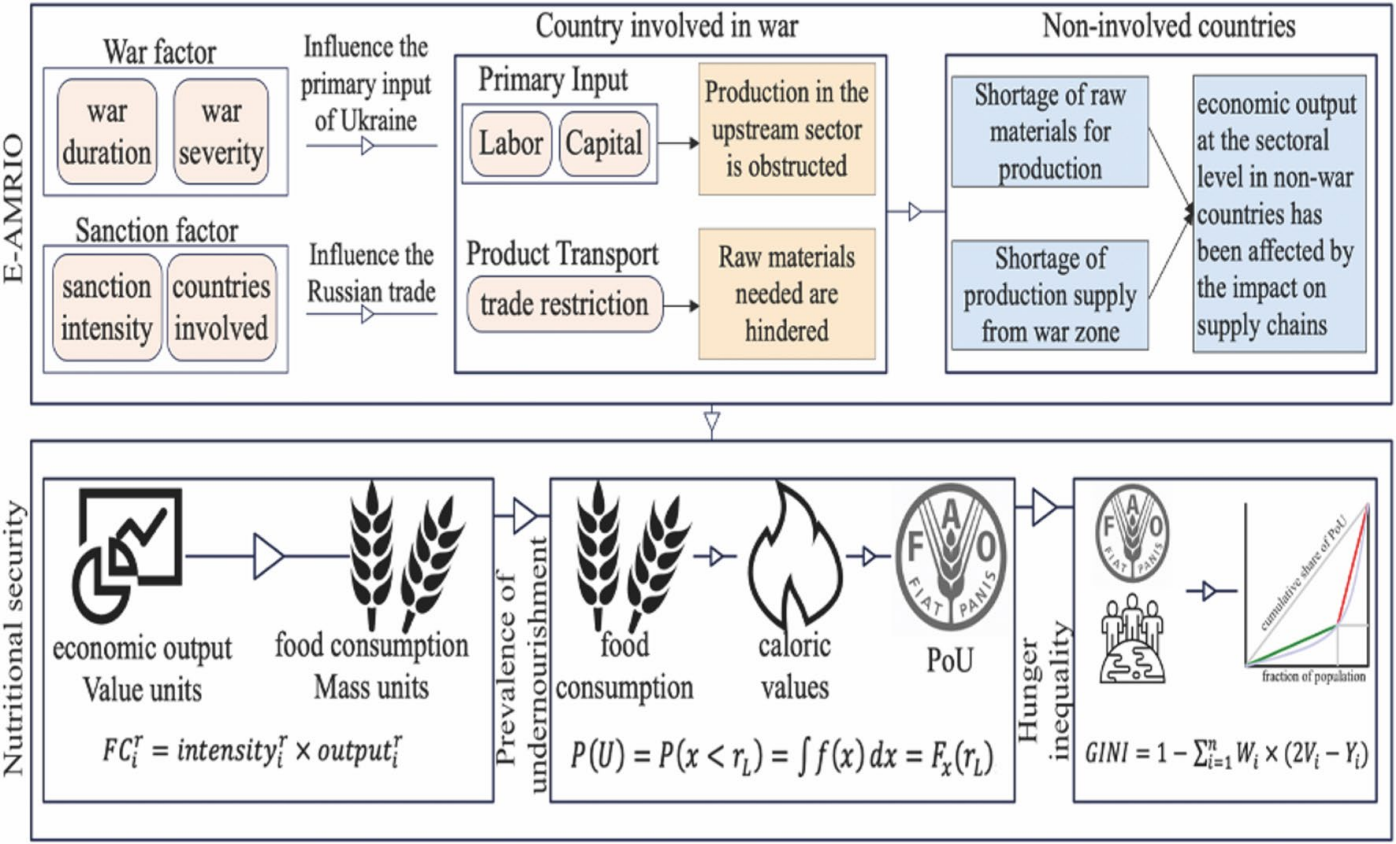
Overview of the backbone of the model

We used the Extended Adaptive Multi-Regional Input–Output (E-AMRIO) model to simulate the impact of the Russia–Ukraine war on global PoU. This model is based on an AMRIO model, which has the advantage of quantifying system-wide economic loss, resource consumption, and environmental pollution from short-term shocks such as natural disasters, pandemics, and public accidents [22, 23]. Our model further improves the previous version in two aspects. First, parameters are set and updated to incorporate war-related factors, e.g., war duration and war intensity, into the model, according to the latest available data. Second, various sanction measures against Russia are linked with the model along the supply chain. Therefore, we refer to this adjusted model as the E-AMRIO model. When war occurs, labor, domestic transportation, and capital input in Ukraine are affected, hindering both domestic production and downstream non-involved countries through supply chain networks. Meanwhile, non-involved countries that impose sanctions on Russia may hamper their own production and consumption due to restrictions on raw materials and food trade. In this manner, we can assess the consequences of the Russia–Ukraine war on the total output of different sectors of countries across the globe by considering various levels of the controlling factors (war duration, war intensity, sanction intensity, and the number of countries involved, Fig. 1). It is worth noting that our model uses the latest GTAP ver10 database with 65 sectors, of which 21 are agricultural sectors. The input–output table in the GTAP database could reflect the complex inter-sectoral linkages in global supply chains. A blockage in any sector of the global supply chain will lead to cascading effects on the production and consumption of other sectors. Our model includes four main modules: the Production Module, the Intermediate Input Module, the Labour Supply Module, and the Demand Module. These four modules fully consider the substitutability of sectoral inputs and dynamic changes in global supply chains (113 countries in total), which more realistically reflect the bottlenecks in global supply chains caused by the war. With the measurement of the supply chain, we can assess economic output at the sectoral level of different countries in different war trajectories. A complete description of mathematical equations can be found in Additional file 1.

### Scenario setting

We consider four important war-related factors: war duration, war severity, sanction intensity, and the number of countries involved [24]. As of September 2022, the Russian-Ukrainian war has been going on for 7 months, and there is no sign of stopping; given the current situation, we have made four assumptions about the duration of the war, that is 9 months, 12 months, 18 months and a particularly extreme 24 months; In addition, due to the difficulty in obtaining available labour in Ukraine and the extent to which domestic transport is affected by the war, We make assumptions about the severity of the war based on the 2022 FAO assessment of the unharvestable area in Ukraine (about 20%) [25], which is a 20% (low), 30% (middle), or 40% (high) reduction in labour availability and transport capacity in Ukraine; For the number of countries involved, we refer to the "Foreign states and territories committing unfriendly ACTS against the Russian Federation, Russian legal entities and individuals" issued by Russia [24], and assume that as the war continues, more countries will participate in the sanctions (Table 1). A total of 108 scenarios were used to simulate the impact of the war on food security, to encourage meaningful discussions.

**Table 1:** Scenario factor assumptions of the Russia–Ukraine war

Factors	Extent	Explanation
War duration	${\mathrm{T}}_{\mathrm{L}}$	The war is expected to last 9 months
	${\mathrm{T}}_{\mathrm{M}}$	The war is expected to last 12 months
	${\mathrm{T}}_{\mathrm{H}}$	The war is expected to last 18 months
	${\mathrm{T}}_{\mathrm{E}}$	The war is expected to last 24 months
War severity	${\mathrm{W}}_{\mathrm{L}}$	The war leads to a 20% reduction in labour availability and transport capacity in Ukraine compared to pre-war levels
	${\mathrm{W}}_{\mathrm{M}}$	The war leads to a 30% reduction in labour availability and transport capacity in Ukraine compared to pre-war levels
	${\mathrm{W}}_{\mathrm{H}}$	The war leads to a 40% reduction in labour availability and transport capacity in Ukraine compared to pre-war levels
Sanction intensity	${\mathrm{S}}_{\mathrm{L}}$	20% limit on exports from “non-war” countries to Russia
	${\mathrm{S}}_{\mathrm{M}}$	50% limit on exports from non-war countries to Russia
	${\mathrm{S}}_{\mathrm{H}}$	80% limit on exports from non-war countries to Russia
Countries involved	${\mathrm{C}}_{\mathrm{L}}$	Participation of countries on the unfriendly list in sanctions against Russia [24]
	${\mathrm{C}}_{\mathrm{M}}$	All countries except Eurasian Economic Union and Shanghai Cooperation Organisation countries are involved in sanctions against Russia [26]
	${\mathrm{C}}_{\mathrm{H}}$	All countries except Russia itself are involved in sanctions against Russia

War duration is classified as low, medium, high, or extreme

### Calculation of food consumption

We calculate the food consumption of each country by summing up its consumption on kinds of agricultural sectors over the supply chain. Limited by data availability, we only consider 15 agricultural sectors here (except raw milk and processed rice), which supply almost all daily nutritional needs. In addition, we add up agricultural products from both domestic and international markets to integrate the impacts of the global supply chain. As a result, we can quantify the food consumption $${FC}_{i}^{r}$$ of country $$r$$ on agricultural sector $$i$$ by multiplying its $${output}_{i}^{r}$$ (unit: million $) and consumption intensity $${int}_{i}^{r}$$ (unit: ton/million $) as following Eq. (1):

1 $${FC}_{i}^{r}={int}_{i}^{r}\times {output}_{i}^{r}$$

### Prevalence of undernourishment (PoU)

We calculated the PoU of each country according to the FAO method [27], which determines the proportion of the population whose caloric consumption does not meet minimum energy requirements:

2 $$P\left(U\right)=P\left(x<{r}_{L}\right)=\int f\left(x\right)dx={F}_{x}\left({r}_{L}\right),$$

where $P\left(U\right)$ is the proportion of undernourished people among the total population, $\left(x\right)$ is the dietary energy, ${r}_{L}$ is the cut-off point reflecting the minimum energy requirement, $f(x)$ is the density function of dietary energy consumption, and ${F}_{x}$ represents the cumulative distribution function. Therefore, we need to convert the food quantity into an energy value, as follows:

3 $$x=FC\times WC\times CC,$$

where $FC$ is food consumption (the total output obtained by the E-AMRIO model can be used to calculate the food consumption in each country, $WC$ is the waste coefficient, and $CC$ is the caloric coefficient. According to Eq. (3), we can convert physical food consumption into caloric values. It is noteworthy that PoU is an indicator on a national scale. Regional or global PoU, in this study, is calculated by dividing the sum of the undernourished population in regional/global by the total regional/global population. We use percentage point as the dedicated unit for PoU in this study to avoid misunderstandings with other occasions where % is used. For example, if the global PoU under scenario A is 4 percentage points, while the global PoU under scenario B is 3 percentage points, then the PoU of scenario A increases by 33.33% compared to scenario B.

### GINI coefficient for food consumption

We quantify hunger inequality with the GINI index. The GINI index is a measure of the distribution of caloric consumption across a population [28]. The GINI index is calculated as:

4 $$GINI=1-\sum_{i=1}^{n}{W}_{i}\times \left(2{V}_{i}-{Y}_{i}\right),$$

Where n is the number of regions, ${W}_{i}$ is the proportion of each country's population in the global population, ${V}_{i}$ is the Cumulative percentage in ascending order of PoU of each country, and ${Y}_{i}$ is the proportion of each country’s PoU in the global PoU. The framework of the model is shown in Fig. 1.

### Data source

We collected base-year macroeconomic and sectoral data from the GTAP v.10 database. We linearly estimated the global population in 2022 using the 2020–2025 population data, under the SSP2 path published by the International Institute for Applied Systems Analysis (IIASA) (www.iiasa.ac.at/). Income groups were divided according to the gross domestic product data published by the World Bank (https://data.worldbank.org/). We collected mean dietary energy consumption and minimum dietary energy requirement data from the FAOSTAT database (http://www.fao.org/faostat/) to calculate the prevalence of undernourishment.

## Result

### Extreme war-induced exacerbation of undernourishment in low-income countries

**Fig. 2:**
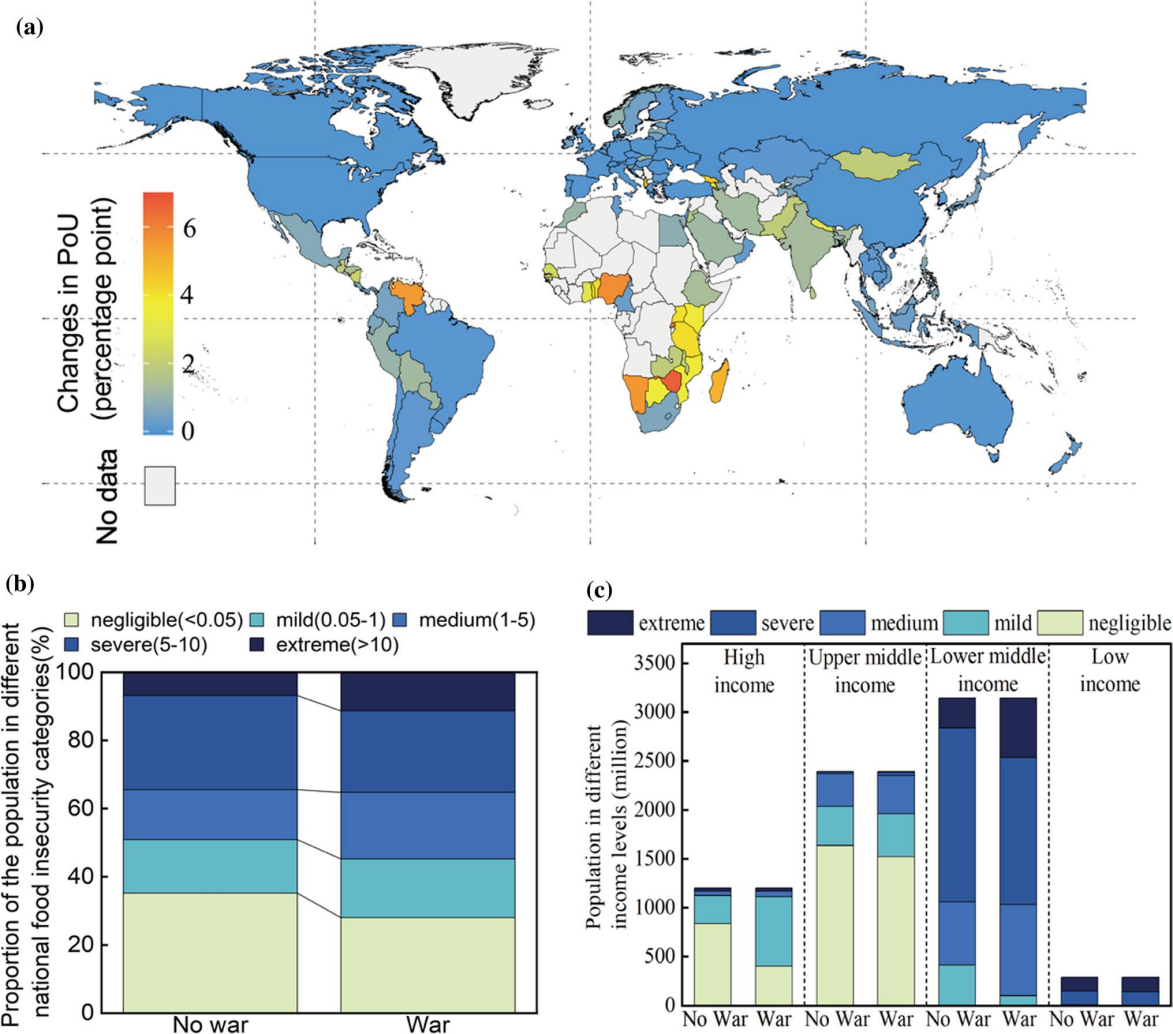
Impact of the Russia–Ukraine war on the global prevalence of undernourishment (PoU) in ${\mathrm{W}}_{\mathrm{L}}{\mathrm{S}}_{\mathrm{L}}{\mathrm{C}}_{\mathrm{L}}{\mathrm{T}}_{\mathrm{L}}$ scenario. **A** Changes in PoU relative to the “No-war” scenario; **b** changes in the proportions of the population in different national PoU categories; **c** the unequal impact of war on countries with different incomes. National food insecurity is categorised into five levels: negligible, mild, medium, severe, and extreme

We find that the Russia–Ukraine war will significantly increase the PoU in almost all countries, especially in the low-income and developing countries in Global South. Taking a situation where the war lasts for 9 months (the ${\mathrm{W}}_{\mathrm{L}}{\mathrm{S}}_{\mathrm{L}}{\mathrm{C}}_{\mathrm{L}}{\mathrm{T}}_{\mathrm{L}}$ scenario) as an example (Fig. 2a), the global PoU would be 4.11 percentage points, which corresponds to an average increase in the global PoU by 30.48% (or an additional 67.32 million people falling into the undernourished category, roughly equivalent to the total population of France), compared to the “No war” scenario (PoU is 3.15 percentage point; see Additional file 2: Table S1 for PoU of each country).

Notably, PoU is an income-related variant; the lower the income of a country, the higher its PoU (using a Gini coefficient among countries of 0.73; see Additional file 2: Table S5). The PoU of low-income countries would change from 15.55 percentage points to 18.32 percentage points, and the increase in PoUs is more than eight times that of high-income countries (change from 0.57 percentage points to 0.89 percentage points). Specifically, African countries, especially low-income ones such as Zimbabwe and Rwanda, are the most vulnerable, with an increase in the PoU of more than 5.50 percentage points in each country. European and North American countries continue to show a relatively stable PoU, with a slight overall increase of 0.07 percentage points. In regions in which countries show a wide range of incomes, such as Asia, the spatial pattern of the PoU varies considerably. Here, South Asia (1.39 percentage points) is the most affected and East Asia (0.06 percentage points) is the least affected area. In short, the PoU will increase in developing countries, especially, the most underdeveloped ones will be most severely affected by the war (see Additional file 2: Table S6 for the PoU of each country under the 108 scenarios).

Our results are much higher than the FAO projections on June, which predicted 11–19 million additional people would fall into undernourishment in the next year. FAO’s projections only accounted for the impact of the food export reduction by Ukraine and Russia. The indirect impacts of disturbance of global supply chains due to the sanctions, labour shortage, and dependence of agricultural production on upstream and downstream products were not considered. For example, Guan, et al. [18] point out that a strict lockdown to contain the COVID-19 outbreak to China would not only cost China 21% of its GDP, but would also affect the rest of the world through global supply chains, reducing global GDP by 3.5%. Our study reveals consistent findings with FAO’s results that Africa, the Middle East and South Asia are heavily affected while highlights the indirect impacts via the global supply chain disturbance.

Due to the different levels of food security faced by countries, we artificially divide the PoUs into five levels: extreme (PoU > 10), severe (10 > PoU > 5), medium (5 > PoU > 1), mild (1 > PoU > 0.05), and negligible (0.05 > PoU). While not all countries are severely affected, those currently experiencing extreme PoUs will inevitably be further affected in terms of their access to food. Therefore, we further analyzed proportion of the population under different levels of PoUs (Fig. 2b). The proportions of the population in negligible PoUs would decline (from 35.20 to 27.98%), bringing the otherwise negligible PoUs to mild PoUs (from 15.69 to 17.22%, see Additional file 2: Table S2). In comparison, six additional countries (including Botswana, Togo, Nepal, Albania, Kenya, and Pakistan) were projected to experience extreme PoUs, such that 316.71 million people (roughly equivalent to the total population of Bangladesh and Russia) would experience more difficulties in accessing food, mostly in lower- and middle-income countries (total population of 303.63 million, of whom 7.36 million would experience undernourishment) and low-income countries (total population of 7.52 million, of whom 0.52 percent points would experience undernourishment). Eight more countries, including Benin, Guinea, Georgia, Trinidad and Tobago, Armenia, Paraguay, Honduras, and Mauritius, were projected to experience severe PoUs (population of 50.88 million). These results show that more people will suffer from extreme hunger due to the war, with low-income and developing countries more severely affected (Fig. 2c, Additional file 2: Table S3).

### Global hunger and inequality associated with longer and more severe war and sanction

**Fig. 3:**
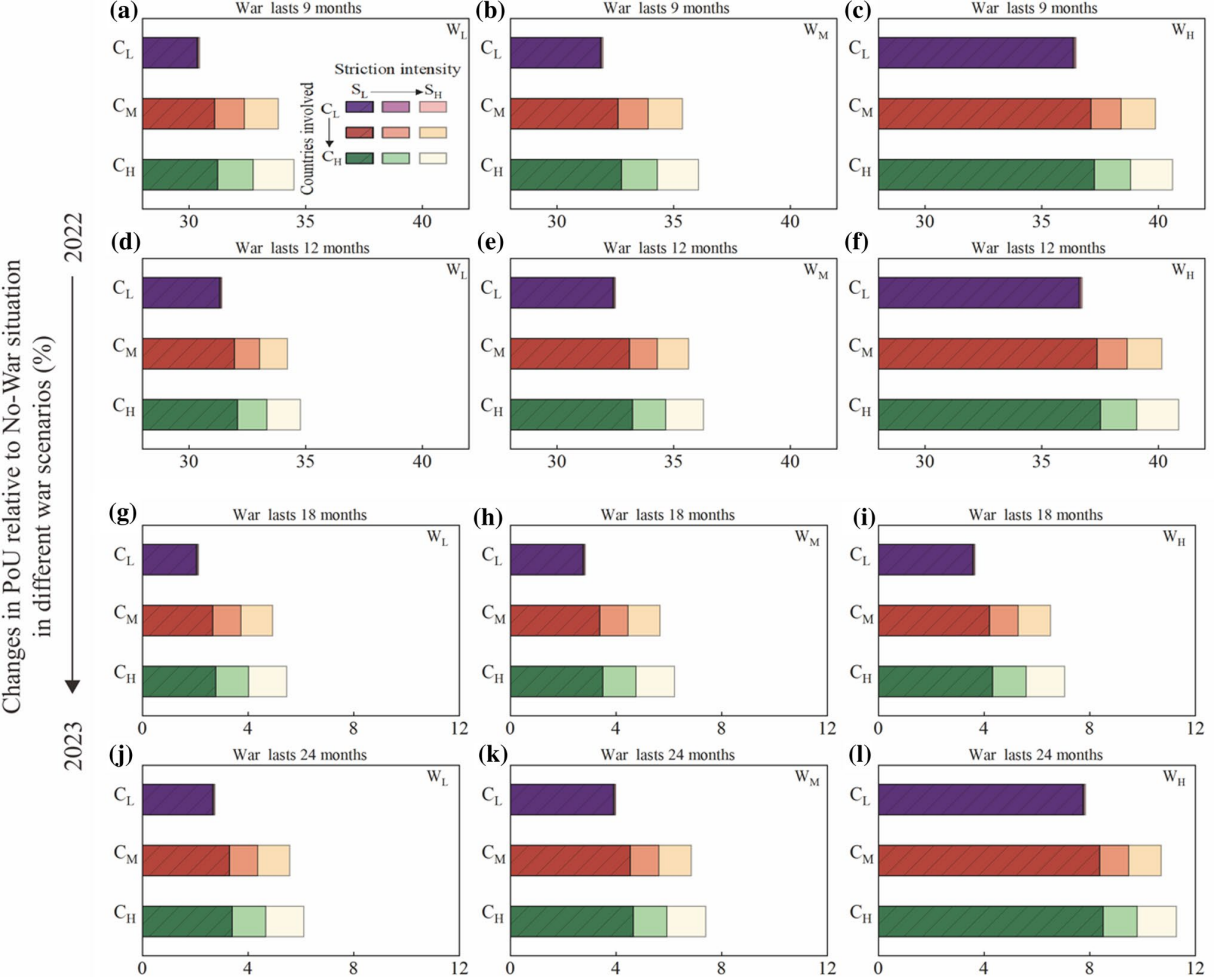
Growing global PoU along with the duration(T), severity(W), sanction(S) intensity, and the number of countries involved (C). The letters L, M and H mean war levels of low, medium, and high. Each bar presents the PoU impacted by one scenario combined with four war factors. A total of 104 scenarios are presented. The panel within **a** indicates the impact of sanctions on global PoU under the same war situation in different colors

Figure 3 shows the results of 108 scenarios, including scenarios of war duration within a year (${\mathrm{T}}_{\mathrm{L}}$ and ${\mathrm{T}}_{\mathrm{M}}$), and scenarios of war duration extended to 2023 (${\mathrm{T}}_{\mathrm{H}}$ and ${\mathrm{T}}_{\mathrm{E}}$). As the war and sanctions continue and worsen, PoU increases in all scenarios. First, the PoU within a year shows an increasing trend as the war continues, even in the lowest severity/response scenario (${\mathrm{W}}_{\mathrm{L}}{\mathrm{S}}_{\mathrm{L}}{\mathrm{C}}_{\mathrm{L}}$; Fig. 3a). As the war duration increases from ${\mathrm{T}}_{\mathrm{L}}$ to ${\mathrm{T}}_{\mathrm{M}}$, the global average PoU would respectively increase by 30.34% and 31.32% compared with the No-war scenario, which could newly place 67.32, and 69.49 million people in undernourishment. Under the worst severity/response scenario (${\mathrm{W}}_{\mathrm{H}}{\mathrm{S}}_{\mathrm{H}}{\mathrm{C}}_{\mathrm{H}}$; Fig. 3c), global nutritional security deteriorates with a protracted war. The global PoU would increase from 40.62% (90.11 million people) under ${\mathrm{W}}_{\mathrm{H}}{\mathrm{S}}_{\mathrm{H}}{\mathrm{C}}_{\mathrm{H}}{\mathrm{T}}_{\mathrm{L}}$ to 40.88% (90.70 million people) under ${{\mathrm{W}}_{\mathrm{H}}{\mathrm{S}}_{\mathrm{H}}{\mathrm{C}}_{\mathrm{H}}\mathrm{T}}_{\mathrm{M}}$. We find that under high war severity, global PoU will increase dramatically with the war lasting only a few months longer.

The long-term impact of the war on PoU, for example in 2023, will be slightly alleviated by the agricultural growth in other non-conflict countries (Fig. 3g). However, the global PoU will not recover to the level of the No-war scenario. For example, the PoU reaches 3.22 percentage points under ${\mathrm{W}}_{\mathrm{L}}{\mathrm{S}}_{\mathrm{L}}{\mathrm{C}}_{\mathrm{L}}{\mathrm{T}}_{\mathrm{H}}$, and increases to 3.24 percentage points under ${\mathrm{W}}_{\mathrm{L}}{\mathrm{S}}_{\mathrm{L}}{\mathrm{C}}_{\mathrm{L}}{\mathrm{T}}_{\mathrm{E}}$, higher than that of the No-war scenario (an increase of 2.04% and 2.67%, respectively). Compared with ${\mathrm{W}}_{\mathrm{L}}{\mathrm{S}}_{\mathrm{L}}{\mathrm{C}}_{\mathrm{L}}{\mathrm{T}}_{\mathrm{M}}$, the agricultural growth mitigates 0.93 percentage points of the global PoU under ${\mathrm{W}}_{\mathrm{L}}{\mathrm{S}}_{\mathrm{L}}{\mathrm{C}}_{\mathrm{L}}{\mathrm{T}}_{\mathrm{H}}$, with developing countries benefiting the most (PoU would fall 0.25 percentage points in developed countries and 1.06 percentage points in developing countries). However, given their already dire PoU situation, agricultural growth will not sufficiently address hunger in developing countries. The production of winter crops and the 2023 crop will also face severe uncertainty if the war is prolonged. Although Ukraine has taken steps to ensure that agricultural activities are staffed, if the war is further prolonged, the number of agricultural laborers at all stages of the supply chain will inevitably be further affected, exacerbating global hunger. The results emphasize that the impact of war on global food security will be lasting in the context of prolonged conflict (see Additional file 2: Table S4).

War severity is a sensitive factor to aggravate PoU. In high severity scenarios, the PoU will substantially increase even if the war duration lasts slightly longer. Direct damage caused by war, labour shortages, and disruptions in the transportation of materials are the main reasons for this to happen. Compared with the No-war scenario, the PoU increases by 31.86% (70.69 million people) under ${\mathrm{W}}_{\mathrm{M}}{\mathrm{S}}_{\mathrm{L}}{\mathrm{C}}_{\mathrm{L}}{\mathrm{T}}_{\mathrm{L}}$ (Fig. 3b), and increases by 36.35% (80.66 million people) under ${\mathrm{W}}_{\mathrm{H}}{\mathrm{S}}_{\mathrm{L}}{\mathrm{C}}_{\mathrm{L}}{\mathrm{T}}_{\mathrm{L}}$ (Fig. 3c) when war severity increases from low to high. Apart from destroying assets (such as roads, bridges, and airports, which are vital to transportation and food supplies) and crops, a more severe and protracted war would reduce capital and labour inputs to agriculture industries in Russia and Ukraine. A sustained and severe war will also disrupt the international grain market, given that about 50 countries currently rely on imports from Russia and Ukraine for > 30% of their wheat supplies [17]. For these countries, the food security situation is particularly concerning.

The global PoU will only slightly increase if more countries are involved in the sanctions, but the impact will be more substantial when sanctions are compounded with the direct impact of the war. As indicated in Fig. 3a, increasing the number of countries involved in sanctions only marginally increases PoU under lower war intensity and duration conditions. More countries evolved in sanctions would increase the PoU as the war continues. For example, the global PoU of ${\mathrm{W}}_{\mathrm{L}}{\mathrm{S}}_{\mathrm{L}}{\mathrm{C}}_{\mathrm{M}}{\mathrm{T}}_{\mathrm{L}}$ increases by 0.75% (1.67 million people) relative to ${\mathrm{W}}_{\mathrm{L}}{\mathrm{S}}_{\mathrm{L}}{\mathrm{C}}_{\mathrm{L}}{\mathrm{T}}_{\mathrm{L}}$; also, the global PoU of ${\mathrm{W}}_{\mathrm{L}}{\mathrm{S}}_{\mathrm{L}}{\mathrm{C}}_{\mathrm{M}}{\mathrm{T}}_{\mathrm{M}}$ relative to ${\mathrm{W}}_{\mathrm{L}}{\mathrm{S}}_{\mathrm{L}}{\mathrm{C}}_{\mathrm{L}}{\mathrm{T}}_{\mathrm{M}}$ increases by 0.64% (1.41 million people). The impact of sanction intensity on PoU also follows the same pattern. For example, the PoU of ${\mathrm{W}}_{\mathrm{L}}{\mathrm{S}}_{\mathrm{H}}{\mathrm{C}}_{\mathrm{L}}{\mathrm{T}}_{\mathrm{L}}$ (Fig. 3a) increases by 0.12% (0.26 million people) compared with ${\mathrm{W}}_{\mathrm{L}}{\mathrm{S}}_{\mathrm{L}}{\mathrm{C}}_{\mathrm{L}}{\mathrm{T}}_{\mathrm{L}}$; And the PoU of ${\mathrm{W}}_{\mathrm{L}}{\mathrm{S}}_{\mathrm{H}}{\mathrm{C}}_{\mathrm{L}}{\mathrm{T}}_{\mathrm{M}}$ would increase by 0.10% (0.21 million people) compared with ${\mathrm{W}}_{\mathrm{L}}{\mathrm{S}}_{\mathrm{L}}{\mathrm{C}}_{\mathrm{L}}{\mathrm{T}}_{\mathrm{M}}$ (Fig. 3d). Our results support that if the sanctions cannot accelerate the termination of the war, they would expose countries that were heavily dependent on Russian–Ukrainian food and energy exports to a more severe demand gap, worsening the welfare of the entire world [3, 29].

**Fig. 4:**
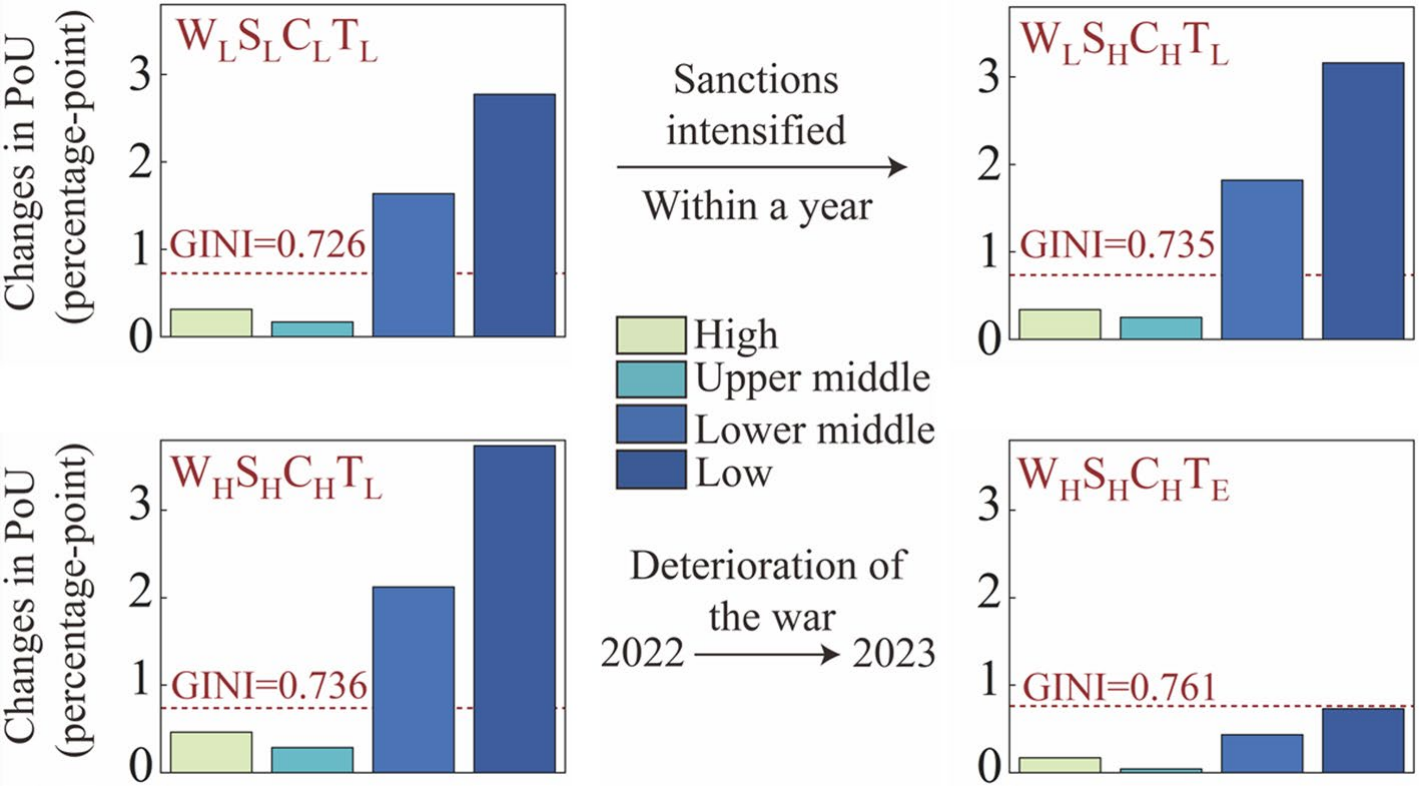
Changes in PoU of different income groups in each scenario relative to the no-war scenario. Each bar presents the PoU of different income groups impacted by one scenario combined with four war factors. The dotted line represents the GINI coefficient under each scenario. A total of 4 scenarios are presented (The reof the results refer to Additional file 2: Table S5)

Our findings also highlight the global inequality in hunger. In all scenarios, low- and lower-middle-income countries are most affected by the war, exposing these countries with high PoUs to more significant food security challenges. For example, under the most optimistic scenario (${\mathrm{W}}_{\mathrm{L}}{\mathrm{S}}_{\mathrm{L}}{\mathrm{C}}_{\mathrm{L}}{\mathrm{T}}_{\mathrm{L}}$), the PoU of lower-middle- and low-income groups changed from 5.70 and 15.55 percentage points under the “No-war” scenario to 6.70 and 18.32 percentage points, an increase of 1.64 and 2.78 percentage points respectively. By contrast, changes in PoU of upper-middle- and high-income groups were small (up 0.18 and 0.32 percentage points, respectively). As can be seen from Fig. 4, with the aggravation of the war and sanctions, the gap between the PoU of the upper-middle- and high-income groups and the lower-middle-income and low-income groups will further increase. For example, under the ${\mathrm{W}}_{\mathrm{L}}{\mathrm{S}}_{\mathrm{H}}{\mathrm{C}}_{\mathrm{H}}{\mathrm{T}}_{\mathrm{L}}$ scenario, the PoU of the low-income group would be up 3.16 percentage points, which is more than 9 times that of the high-income group (up 0.34), making inequality even more severe.

To further measure the existence of inequality, we introduce the GINI coefficient for verification. The results show that the inequality represented by the Gini coefficient is exacerbated as the Russia–Ukraine war drags on. From the most optimistic scenario (${\mathrm{W}}_{\mathrm{L}}{\mathrm{S}}_{\mathrm{L}}{\mathrm{C}}_{\mathrm{L}}{\mathrm{T}}_{\mathrm{L}}$) to the most pessimistic scenario (${\mathrm{W}}_{\mathrm{H}}{\mathrm{S}}_{\mathrm{H}}{\mathrm{C}}_{\mathrm{H}}{\mathrm{T}}_{\mathrm{E}}$), the Gini coefficient increases from 0.728 to 0.761(Additional file 2: Table S5). Moreover, the Gini coefficient will continue to increase as more severe sanctions and war (with ${\mathrm{S}}_{\mathrm{H}}$ and ${\mathrm{C}}_{\mathrm{H}}$ being the most severe) are imposed. For example, the GINI coefficient would increase from 0.726 under the ${\mathrm{W}}_{\mathrm{L}}{\mathrm{S}}_{\mathrm{H}}{\mathrm{C}}_{\mathrm{H}}{\mathrm{T}}_{\mathrm{L}}$ scenario to 0.735. However, under the same sanctions’ conditions, the intensification of war factors (T and W) would reduce the GINI coefficient. For example, the GINI coefficient would decrease from 0.726 under the ${\mathrm{W}}_{\mathrm{L}}{\mathrm{S}}_{\mathrm{L}}{\mathrm{C}}_{\mathrm{L}}{\mathrm{T}}_{\mathrm{L}}$ scenario to 0.715 under the ${\mathrm{W}}_{\mathrm{H}}{\mathrm{S}}_{\mathrm{L}}{\mathrm{C}}_{\mathrm{L}}{\mathrm{T}}_{\mathrm{L}}$. Besides, the GINI coefficient would change from 0.736 under the ${\mathrm{W}}_{\mathrm{H}}{\mathrm{S}}_{\mathrm{H}}{\mathrm{C}}_{\mathrm{H}}{\mathrm{T}}_{\mathrm{L}}$ scenario to 0.735 under the ${\mathrm{W}}_{\mathrm{H}}{\mathrm{S}}_{\mathrm{H}}{\mathrm{C}}_{\mathrm{H}}{\mathrm{T}}_{\mathrm{M}}$, but this does not mean that global food inequality has been alleviated. This emphasizes that when the war continues, countries in all income groups would face more severe food crises (see Additional file 2: Table S5).

Heterogeneity in hunger among countries is already severe due to the COVID-19 pandemic, and the war will make it even harder for developing countries to access enough food. Therefore, synthesizing different extents of the impact of the war and heterogeneity in hunger, and formulating measures to reduce PoU in developing countries is crucial to cope with the shocks of the war on well-being.

## Discussion

### Vulnerability in middle- and low-income countries

Our results, based on enumerative modeling of various war trajectories, measured the global vision of degressive nutritional security. We highlight that low- and middle-income countries will suffer from a much larger increase of PoU than high income ones in all scenarios. This shows that the Russian-Ukrainian war will do far more harm to low- and middle-income countries that were not directly involved in the war than to high income countries. This is mainly due to the lower agricultural production capacity, grain reserves, and poor resistance to international food price fluctuations in such countries, etc [30, 31]. Given the large population in the low- and middle-income countries and the existing high levels of undernourishment, more global attention should be paid to those countries. Children and women in low- and middle-income countries would be the priority to maintain a nutritious diet, due to their vulnerability to health risks [32, 33].

### War duration and severity are crucial factors

Changes in PoU are more sensitive to war duration and severity than the other two factors, that is, sanctions and number of countries involved. In addition, it seems that tougher sanctions and more countries participating in the sanctions would not stop the war, but would make the participating countries face more food and energy supply shortages; also, the abuse of sanctions will only lead to more intensified conflicts between countries. Military and other support for Ukraine by the United States, European countries, Japan, etc. also makes the duration and severity of the Russian-Ukrainian war more difficult to predict. In order not to let the war continue to worsen, the international community and direct stakeholders should be more active and start all possible peace negotiations as soon as possible, so as to end the threat of war to food supply and life security [34].

### Boost food accessibility

Our results show that the increased PoU will be only slightly alleviated, even if agriculture production recovered. Low-income and food-deficit countries that rely heavily on imports from Ukraine and Russia to meet their food needs (such as Armenia and Rwanda) are the most vulnerable nations. These countries should actively adjust their dietary structure, starting from the lowest calorie requirement and reducing their dependence on a single food. In addition, the yield gap can be actively closed through sustainable agricultural intensification methods to increase the country’s food self-sufficiency [35]. For many African countries, closing the yield gap is not enough, these countries should actively adjust their trade structure [36] and establish trade partnerships with different countries to ensure sufficient food supply and consumption. Diversified sources of food supplies could help these countries withstand the shock of war. However, more food supply chains may lead to increased greenhouse gas emissions associated with food transport [37, 38]; additionally, other countries may expand arable land to alleviate food security in order to compensate for the decline in food imports, which may come at the expense of potentially large carbon emissions [39]. Meanwhile, due to the impact of war, the price of food and agricultural inputs has also continued to rise, restricting the public to obtain enough food and exacerbating the PoU. Therefore, strengthened subsidies on food and agricultural inputs with soaring prices, such as fertilizer and fuel, would be an essential safeguard for these countries to alleviate PoU [40]. For example, about a quarter of imported fertilizer in Brazil and Mexico comes from Russia.

### International collaborations are critical to “zero hunger” goal

Food trade restrictions, including sanctions and export bans, should be released to restore the global food supply chain. International organizations such as the FAO and the WHO should collaborate on response plans to provide agricultural assistance to the countries most affected by the war and ensure their food security [41, 42]. Consuming a healthier diet with less meat in North America and Europe would enable more compensation of the food loss, and meanwhile, benefit the climate mitigation and biological conservation. Also, major grain-producing countries and high income countries should be encouraged to increase targeted exports to high PoU countries, to stabilize food prices and ensure basic food intake needs are met [43].

The recent agreement between Russia and Ukraine to keep food shipments flowing in Turkey [43] helps reduce the risk of global food shortages. However, due to the high level of uncertainty over the trajectory of the war, the smooth implementation of future agreements is still highly influenced by the development of the war. By contrast, if the war is prolonged, both the nutrition security of developing and industrialized countries will still be exposed to this high level of uncertainty. As a result, whether the UN’s Zero Hunger” goal by 2030 can be achieved remains highly doubtful. Under such circumstances, it will be essential to confine the war in terms of severity and geographical scope, to reduce the loss of labour and land for agriculture and seize the “window of opportunity” for spring planting to alleviate the supply shortage [42]. Therefore, stakeholders, especially governments in developing countries, should develop a package plan for lower PoU in different war scenarios.

## Limitations

A key limitation of the study is the enumerable scenario settings. We listed a wide range, but still unexhausted cases of future hypothetical future trajectories of the war to investigate how the war could affect global hunger. However, due to the rapidly changing forms of warfare, it’s almost an impossible task to predict the impact of the Russian–Ukrainian war dynamically and accurately.

Our model is primarily based on current trade relations in the global economy and does not consider changes in the production mix or economic structure. Wars and sanctions could further increase food prices, putting even more people at risk due to paralyzed supply chains. In addition, the model didn’t simulate changes in global energy prices, fertilizer production and trade affected by the wars and sanctions [31]. However, it must be acknowledged that due to the complexity of the non-optimal nature of economies, it remains difficult to fully quantify this uncertainty in simulations. Even with the above limitations, our model has clearly revealed the apparent unequal impacts on developing countries. We expect this inequality will be higher when price and structural changes are considered, given the weaker capability of developing countries to hedge against such shocks. Thus, future work should focus on these aspects when estimating the impact of short-term shocks on PoU.

## Supplementary Information

**Additional file 1: **The Russia–Ukraine war disproportionately threatens the nutrition security of developing countries.

**Additional file 2: Table S1.** Changes in PoU in each countries relative to the “No-war” situation. **Table S2.** Total demographic changes at different levels of national food insecurity (million people). **Table S3.** demographic changes in countries at different levels of national food insecurity (million people). **Table S4.** Changes in PoU relative to No-war scenario in different war scenarios (%) and newly place people in undernourishment (million people). **Table S5**. Gini coefficients under 108 scenarios. **Table S6**. PoU in each country under different war scenarios (Unit: percentage point).

## Data Availability

Data supporting the findings of this study are available within the article and its Supplementary Information files or are available from the corresponding author upon reasonable request.

## Code availability

The codes developed for the analyses and to generate results are available from the corresponding author on reasonable request.
