# Supplementary material for: The Russia–Ukraine war disproportionately threatens the nutrition security of developing countries
Source: Discov Sustain. 2022 Nov 17;3(1):40. doi: 10.1007/s43621-022-00112-8 (PMC9670081; doi:10.1007/s43621-022-00112-8)
Supplement: Supplementary file 1 — Additional file 1: The Russia–Ukraine war disproportionately threatens the nutrition security of developing countries. [file 43621_2022_112_MOESM1_ESM.pdf]

Supplementary information for
The Russia–Ukraine war disproportionately threatens the nutrition security of developing countries

Deng et al.

This section provides supplementary methods for the main text.

We use a global adaptive multiregional input-output (AMRIO) model[1,2] to evaluate the shocks of the Russian-Ukrainian war on global food supplies, that caused cascading effects on the prevalence of undernourishment in each country. The AMRIO model is one of the most effective tools to quantify the economic loss, resources consumption and environmental pollution derived from the disaster such as the pandemic, and public service disruption[3-6]. Its advantage is accounting for interactions between supply and demand at the sectorial level based on a global input-output table, in the meanwhile, flexibility in adjusting to short term shocks from the disaster impacts[4,7]. The AMRIO model uses the latest available global input-output data (GTAP10)[8] and accounts for interactions between supply and demand at the sectoral level. Different from other models such as computable general equilibrium (CGE) models, this model run on weekly time steps, which is particularly useful for evaluating short-term contingencies relative to models that are typically run on annual time steps.

The AMRIO model contains four main modules: the Production Function Module, the Intermediate Input Module, the Labor Supply Module, and the Demand Module. Here, we refer to the model proposed by Wang[9] and extend the impact of the Russian-Ukrainian war to four controlling factors: the restrictions on labor and transportation in

Ukraine brought about by the Russian-Ukrainian war, the duration of the Russian-Ukrainian war, the intensity of sanctions in the non-theater zone against Russia and the number of countries involved. According to this, we can assess the consequences of the Russian-Ukrainian war on global grain trade by considering different war factors and trade sanctions. A detailed description of the model structure, equations, parameters and model simulation is provided below.

Production Function Module

There are many estimation methods for industrial production, such as the Leontief production function[10], Cobb-Douglas (C-D) function, and Constant Elasticity of Substitution (CES) function[11]. Considering that there is no prediction about the occurrence of a war, and economic agents cannot adjust in time, this study chooses the Leontief production function. Since it does not allow substitution between inputs, it is most suitable for this study.

According to the Leontief function, the output from sector j in region i ($x_{j,i}$) can be expressed in the following equation:

$$x_{j,i} = min\left(for\ all\ m, \frac{z_{j,i}^{m}}{a_{j,i}^{m}}; \frac{va_{j,i}}{b_{j,i}}\right) \tag{1}$$

where m denotes type of intermediate products; $z_{j,i}^{m}$ refers the intermediate product m used in sector j; $va_{j,i}$ refers the primary inputs for the sector j, including labour and capital. $a_{j,i}^{m}$ and $b_{j,i}$ are the input coefficients of intermediate products m and primary inputs of sector j, which can be calculated in Equation 2. All economic transactions and the interdependence of industries are expressed in value units.

$$a_{j,i}^m = \frac{\bar{z}_{j,i}^m}{\bar{x}_{j,i}}, \ b_{j,i} = \frac{\overline{va}_{j,i}}{\bar{x}_{j,i}} \tag{2}$$

From the perspective of industries, we assume that each industry produces unique goods or service, and need primary inputs, i.e., capital and labor, as well as intermediate inputs from all other industries. If the Russia-Ukraine war has not occurred, the output of each industry is able to meet the demand of households (final demand) and other industries (intermediate input) not only in the local region but also outside the region. However, if the global supply chain is shocked by the Russian-Ukrainian war and the corresponding sanctions, we believe that Labor Supply, Intermediate Inputs, and Demand activities will be reshaped by the Russian-Ukrainian war.

Capital limitation

Due to the decline in available labor and the tightening of the global supply chain, the capital market has also received a serious impact, hindering production activities, making the capital limitation also one of the bottlenecks in production activities ($x_j^{Cap}(t)$).

$$x_j^{Cap}(t) = \frac{Cap_{j,i}(t)}{b_{j,i}} \tag{3}$$

Where $x_j^{Cap}(t)$ refers to the maximum output when the capital market is restricted. $Cap_{j,i}(t)$ the primary inputs for the firm j at time step t.

If we consider the limitations of primary, capital limitation, and intermediate inputs, the maximum production capacity of sector j in time t is :

$$x_{j,i}^{max}(t) = min\left(x_j^{Lab}(t);\ x_j^{Cap}(t);\ for\ all\ j,i,x_{j,i}^{Sup}(t)\right) \tag{4}$$

Supply constraints

In terms of demand, there is a shortage of food production and supply due to the war. Hence, the total order demand for the sector j in period t $(TD_{j,i}(t))$ equals to the sum of intermediate demand and household demand.

$$TOD_{j,i}(t) = \sum_{q,s} FOD_{j,i}^{q,s}(t) + \sum_{s} HOD_{j,i}^{s}(t) \quad (5)$$

where $FOD_{j,i}^{q,s}(t)$ refers the order demand that sector q in region s required from supplier j in region i; $HOD_{j,i}^{s}(t)$ is the order demand that household in region s required from supplier j in region i

Intermediate inputs Module

$The\ mth$ intermediate products at time $t + 1$ in sector j of region i is represented as $\bar{z}_{j,i}^{m}(t + 1)$. It is determined by the inventory in the last time period $Sup_{j,i}^{m}(t)$:

$$\bar{z}_{j,i}^{m}(t + 1) = Sup_{j,i}^{m}(t) \quad (6)$$

$Sup_{j,i}^{m}(t)$ equals the inventory at time $t - 1$ $(Sup_{j,i}^{m}(t - 1))$ minus the usage during time t $(Sup\_used_{j,i}^{m}(t))$ plus the inventory increase during time t $(Sup\_added_{j,i}^{m}(t))$:

$$Sup_{j,i}^{m}(t) = Sup_{j,i}^{m}(t - 1) - Sup_{used}{}_{j,i}^{m}(t) + Sup_{added}{}_{j,i}^{m}(t) \quad (7)$$

The storage of intermediate input at the initial stage $Sup_{j,i}^{m}(0)$ is proportional to (∂t) to the intermediate input:

$$Sup_{j,i}^{m}(0) = \partial \times \bar{z}_{j,i}^{m}(0) \quad (8)$$

Inventory used during time t $(Sup\_used_{j,i}^{m}(t))$ is:

$$Sup_{used}{}_{j,i}^{m}(t) = x_{j,i}^{m}(t) \times a_{j,i}^{m} \quad (9)$$

Inventory added in time t ($Sup\_added^{m}_{j,i}(t)$) is the actual intermediate input allocated to sector i in time t, the $AOF^{m}_{j,i}(t)$:

$$Sup_{added}{}^{m}_{j,i}(t) = AOF^{m}_{j,i}(t) \tag{10}$$

$AOF^{m}_{jq,is}(t)$ is the mth product of sector j in region q allocated to sector i in region s, which is jointly determined by the production of sector j in region q and the proportion of demand of sector i in region s. Assuming that each sector decides the allocation of mth products according to the orders it gets from the last time period:

$$AOF^{m}_{jq,is}(t) = \frac{FOD^{s,q}_{i,j}(t-1)}{\sum_s FOD^{s,q}_{i,j}(t-1)+\sum_s HOD^{s,q}_{h,j}(t-1)} \times x^{q}_{j}(t) \tag{11}$$

where $FOD^{s,q}_{i,j}(t-1)$ represents the order issued by sector q to its supplying sector j at time period $t-1$, $HOD^{s,q}_{h,j}(t-1)$ is measured by the household order demand and the supply capacity of their suppliers. $x^{q}_{j}(t)$ is the production of sector j in region q. Calculation of $FOD^{s,q}_{i,j}(t-1)$ and $HOD^{s,q}_{h,j}(t-1)$ is explained in the Demand Module.

Labor Supply Module

War-induced labor constraints could have serious knock-on effects on food production and beyond. In the context of the Russian-Ukrainian war, the inability of employees to work due to death or war constraints is a key factor to consider when assessing impact disasters. In our model, the proportion of surviving productive capacity from the constrained labor productive capacity (x^{Lab}_{j}) after a shock is defined as:

$$x^{Lab}_{j}(t) = \left(1 - \sigma^{Lab}_{j}(t)\right) \times \bar{x}_{j} \tag{12}$$

Where $\sigma_j^{Lab}(t)$ is the proportion of labor that is unavailable at each period t during containment. $\left(1 - \sigma_j^{Lab}(t)\right)$ contains the available proportion of employment at period t.

$$\sigma_j^{Lab}(t) = \left(\bar{\sigma}_j - \sigma_j(t)\right)/\bar{\sigma}_j \tag{13}$$

The proportion of the available productive capacity of labor is thus a function of the losses from the sectoral labor forces and its pre-disaster employment level. Following the assumption of the fixed proportion of production functions, the productive capacity of labor in each region after a disaster (x_j^{Lab}) will represent a linear proportion of the available labor capacity at each time step.

$$x_{j,i}^{Sup}(t) = \frac{Sup_{j,i}^{m}(t-1)}{a_{j,i}^{m}} \tag{14}$$

Where $x_{j,i}^{Sup}(t)$ refers to the maximum output when the intermediate input product is restricted. $Sup_{j,i}^{m}(t-1)$ means the stock of mth intermediate products in the inventory of firm j at the end of time step $t-1$.

Demand Module

To make a more realistic representation to the real production process, we assume that each sector holds some inventory of intermediate goods. In each time step, sectors use intermediate products from their inventories for production, and purchase intermediate products from their supplying sectors to restore their inventories

The amount of intermediate product m hold by sector q in region s in period t is denoted as $Sup_{q,s}^{m}(t)$, and we assume the inventory of intermediate product m

required by sector q in region s is $Sup_{q,s}^{m,*}(t)$, which could fulfil its consumption for $n_{q,s}^{m}$ days.

$$Sup_{q,s}^{m,*}(t) = n_{q,s}^{m} \times a_{q,s}^{m} \times x_{q,s}^{max}(t) \tag{15}$$

Then the order issued by sector q to its supplying sector j is:

$$FOD_{j,i}^{q,s}(t) = \begin{cases} \left(Sup_{q,s}^{m,*}(t) - Sup_{q,s}^{m}(t)\right) \times \dfrac{\overline{FOD}_{j,i}^{q,s} \times x_{j,i}^{max}(t)}{\sum_{q \to m}\left(\overline{FOD}_{j,i}^{q,s} \times x_{j,i}^{max}(t)\right)}, & if \ Sup_{q,s}^{m,*}(t) > Sup_{q,s}^{m}(t); \\ 0 & if \ Sup_{q,s}^{m,*}(t) \leq Sup_{q,s}^{m}(t). \end{cases} \tag{16}$$

$HOD_{j,i}^{s}(t)$ is measured by the household order demand and the supply capacity of their suppliers. In this study, the demand of final products q by demand of household in region s, $HD_{s}^{q}(t)$, is given exogenously at each time step. Then, the order issued by household s to its supplier j is:

$$HOD_{j,i}^{s}(t) = HD_{s}^{q}(t) * \frac{\overline{HOD}_{j,i}^{s} * x_{j,i}^{max}(t)}{\sum_{j \to q}\left(\overline{HOD}_{j,i}^{s} * x_{j,i}^{max}(t)\right)} \tag{17}$$

Taking both forward effects and backward effects into consideration simultaneously, the actual output of the producer j in period t ($x_{j,i}^{Act}(t)$) is

$$x_{j,i}^{Act}(t) = min\left(x_{j,i}^{max}(t), TOD_{j,i}(t)\right) \tag{18}$$

The actual production will be allocated to downstream economic sectors and households according to their orders. If the output is not enough to meet all orders, the output of a firm will not be able to fulfill all the orders of its clients. A rationing scheme that reflects a mechanism on the basis of which a firm allocates an insufficient amount of products to its clients is needed. Similarly, if the firm's transportation to customers

or suppliers is restricted, production will also be affected. For example, during the outbreak of war between Russia-Ukraine, the authorities imposed a strict war blockade. These measures restrict the supply of labor and the transportation of products. This has led to a reduction in Ukraine's output and has also triggered forward and backward effects.

Food consumption

This section provides supplementary methods on how to calculate food consumption.

Using the multi-region input-output table output by the AMRIO model, we can calculate the weekly total output of the agricultural sector under different war paths:

$$X_{week} = AX_{week} + Y_{week} = (I - A)^{-1}Y_{week} = LY_{week} \tag{19}$$

where I, A, L, and Y_{week} represent the identity matrix, technical coefficient matrix, Leontief inverse matrix, and weekly final demand, respectively. After adding up, we can get the total output of the food sector in a year:

$$X_{total} = \sum_{i=1}^{52} X_{week}^{i} \tag{20}$$

we can estimate food consumption for each country on the basis of consumption-based estimation, referring to their consumption intensity multiplied by economic output:

$$Food_i^r = intensity_i^r \times {X_{total}}_i^r \tag{21}$$

In the equation, $Food_i^r$ indicates the food consumption of sector i in region r. $intensity_i^r$ represents the food consumption per unit of output of sector i in region r before the war (we assume food consumption intensity will not change with war).

1. Z. Zeng, D. Guan, A. E. Steenge, Y. Xia, D. Mendoza-Tinoco, Flood footprint assessment: a new approach for flood-induced indirect economic impact measurement and post-flood recovery. Journal of Hydrology **579**, 124204 (2019).
2. S. Hallegatte, An Adaptive Regional Input-Output Model and its Application to the Assessment of the Economic Cost of Katrina. Risk Analysis **28**, 779–799 (2008).
3. D. Guan, et al., Global supply-chain effects of COVID-19 control measures. Nature Human Behaviour 4, 577–587 (2020).
4. Y. Shan, et al., Impacts of COVID-19 and fiscal stimuli on global emissions and the Paris Agreement. Nature Climate Change 11, 200–206 (2020).
5. D. Wang, et al., Economic footprint of California wildfires in 2018. Nat Sustain 4, 252–260 (2021).
6. Zhang, Y. *et al.* The perceived effectiveness and hidden inequity of postpandemic fiscal stimuli. *Proceedings of the National Academy of Sciences* **119**, e2105006119, doi:doi:10.1073/pnas.2105006119 (2022).
7. Hallegatte, S. Modeling the role of inventories and heterogeneity in the assessment of the economic costs of natural disasters. Risk analysis 34, 152-167 (2014).
8. Aguiar, A., Chepeliev, M., Corong, E., McDougall, R. & Dominique, V. D. M. The GTAP Data Base: version 10. J. Glob. Econ. Anal. 4, 1–27 (2019).

9. Wang, D. DaopingW/economic-impact-model: Disaster Footprint Model (v1.0). Zenodo. https://doi.org/10.5281/zenodo.4290117

10. Koks, E. E. et al. Regional disaster impact analysis: comparing input–output and computable general equilibrium models. Natural Hazards and Earth System Sciences 16, (2016).

11. Miller, R. E. & Blair, P. D. Input–Output Analysis Foundations and Extensions. vol. 64 (Cambridge University Press, 2009).
